# Primary Aldosteronism Presenting as Dropped Head Syndrome With Hypokalemic Rhabdomyolysis: A Case Report

**DOI:** 10.1155/crie/6424516

**Published:** 2026-04-29

**Authors:** Ya-Chen Kao, Yu-Cheng Liang, Ye-Fong Du, Horng-Yih Ou

**Affiliations:** ^1^ Department of Internal Medicine, National Cheng Kung University Hospital, College of Medicine, National Cheng Kung University, Tainan, Taiwan, ncku.edu.tw

**Keywords:** aldosterone-producing adenoma, dropped head syndrome, hypokalemia, primary aldosteronism, rhabdomyolysis

## Abstract

**Background:**

Primary aldosteronism (PA) is a common cause of secondary hypertension and hypokalemia, but it rarely presents with neuromuscular symptoms. Although only a few cases of hypokalemia‐induced rhabdomyolysis have been reported, dropped head syndrome (DHS) due to cervical axial muscle weakness has not previously been described in the context of PA.

**Case Presentation:**

A 65‐year‐old woman with a history of hypertension presented with progressive posterior neck weakness, followed by proximal limb soreness and weakness. Cervical spine disease was initially suspected, but CT imaging revealed only degenerative changes without cord compression. Laboratory tests showed severe hypokalemia (1.5 mmol/L), metabolic alkalosis, and markedly elevated creatine kinase and myoglobin levels, consistent with rhabdomyolysis. Her symptoms resolved after potassium supplementation. Endocrine workup revealed an elevated aldosterone‐to‐renin ratio (ARR) and a positive captopril suppression test. CT and NP‐59 SPECT/CT identified a left adrenal functioning adenoma. Laparoscopic adrenalectomy confirmed an aldosterone‐producing adrenal cortical adenoma. Postoperatively, the patient achieved complete biochemical remission with normalized potassium levels, improved blood pressure, and no recurrence of muscle weakness or neck symptoms.

**Conclusion:**

This case highlights a rare presentation of PA as hypokalemia‐induced rhabdomyolysis and DHS mimicking cervical spine pathology. In patients with unexplained focal muscle weakness and persistent hypokalemia, particularly in the setting of hypertension, PA should be considered. Clinicians should maintain a high index of suspicion and pursue comprehensive diagnostic evaluations. Early diagnosis and definitive treatment can prevent long‐term complications and enable full metabolic recovery.

## 1. Introduction

Primary aldosteronism (PA) is a leading cause of secondary hypertension, results from excessive aldosterone secretion by the adrenal glands [1]. This hormonal excess leads to sodium retention, intravascular volume expansion, and elevated blood pressure. In more severe cases, it may also cause hypokalemia [1].

In rare instances, profound hypokalemia in patients with PA can lead to neuromuscular complications such as muscle weakness, myalgia, or even rhabdomyolysis [2]. These symptoms typically affect proximal muscles, such as the thighs or shoulders, rather than distal or axial muscle groups [3].

Here, we present the first reported case of PA initially manifesting as dropped head syndrome (DHS), a form of cervical axial muscle weakness, mimicking cervical spine disease.

## 2. Case Presentation

A 65‐year‐old woman with a 10‐year history of hypertension, managed with nifedipine, presented to the emergency department with a 1‐week history of progressive posterior neck weakness. She reported difficulty raising her head, followed by soreness and weakness in both thighs that impaired her ability to walk. In the subsequent days, she developed upper arm weakness, although grip strength was relatively preserved. No recent aggravating factors or relevant exposures were reported.

She had no history of smoking, alcohol use, betel nut chewing, or illicit drug use. Her family history was notable for hypertension in her sister, but was otherwise unremarkable. On arrival, her blood pressure was markedly elevated at 195/67 mm Hg. Neurologic examination revealed symmetric proximal muscle weakness (neck extensor: 2/5, bilateral shoulder abduction: 3/5, bilateral hip flexion: 3/5; others: 4–5/5) without sensory deficits. Deep tendon reflexes were generally decreased. Cervical spine CT revealed osteoarthritis of the right C3–C6 facet and uncovertebral joints, without cord compression or acute pathology.

Laboratory studies revealed profound hypokalemia (serum potassium 1.5 mmol/L) and metabolic alkalosis suggested by venous blood gas analysis (pH 7.475, HCO_3_^−^ 35.2 mmol/L). Serum sodium was 140 mmol/L and chloride 93 mmol/L. Renal function was mildly impaired, with a creatinine level of 0.98 mg/dL (estimated glomerular filtration rate 57 mL/min/1.73 m^2^). Liver enzymes showed mild elevation (ALT 82 U/L). Thyroid function tests were within normal limits (TSH 1.37 µU/mL, free T4 1.16 ng/dL, total T3 99.41 ng/dL). Marked elevation of creatine kinase (17,685 U/L) and serum myoglobin (11,350 ng/mL) indicated severe muscle injury with rhabdomyolysis. Urinalysis demonstrated a positive dipstick test for blood (250 cells/µL) with minimal red blood cells on microscopic examination (0–2 per high‐power field), indicating myoglobinuria (Table 1). Electrocardiography revealed T‐wave inversion, ST‐segment depression, and prominent U waves, consistent with severe hypokalemia.

**Table 1:** Key laboratory findings on admission.

Parameter	Result	Reference range
Electrolytes and renal function		
Serum potassium (mmol/L)	1.5	3.5–5.0
Serum sodium (mmol/L)	140	135–145
Serum chloride (mmol/L)	93	98–106
Creatinine (mg/dL)	0.98	0.6–1.2
Estimated GFR (mL/min/1.73 m^2^)	57	≥60
Venous gas analysis		
pH	7.475	7.35–7.45
PCO_2_ (mm Hg)	48.9	35–45
PO_2_ (mm Hg)	32.7	—
Bicarbonate (mmol/L)	35.2	22–26
Base excess (mmol/L)	10.0	−2 to +2
SaO_2_ (%)	66.2	—
Liver function		
Alanine aminotransferase (U/L)	82	<40
Muscle injury markers		
Creatine kinase (U/L)	17,685	<200
Serum myoglobin (ng/mL)	11,350	<85
Thyroid function tests		
Thyroid‐stimulating hormone (µU/mL)	1.37	0.4–4.0
Free thyroxine (ng/dL)	1.16	0.8–1.8
Total triiodothyronine (ng/dL)	99.41	80–200

Abbreviation: GFR, glomerular filtration rate.

**Figure 1:**
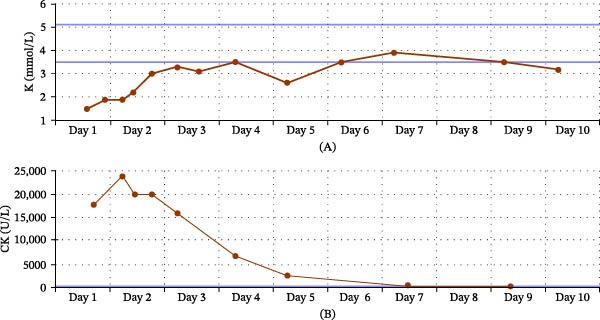
Serial changes in serum potassium and creatine kinase levels during hospitalization. (A) Serum potassium levels progressively increased from a profoundly hypokalemic state on Day 1 to near‐normal levels with potassium replacement therapy. (B) Serum creatine kinase (CK) levels markedly elevated at presentation, consistent with rhabdomyolysis, and gradually declined with correction of hypokalemia and hydration.

Under the diagnosis with hypokalemic rhabdomyolysis and hypertension, she received intravenous fluid resuscitation, potassium supplementation, and antihypertensive therapy. After the above management, her serum potassium level increased to 3.1 mmol/L, and her creatine kinase level decreased to an undetectable level (Figure 1). The patient achieved full recovery of muscle strength without residual weakness and was subsequently discharged.

To investigate the underlying cause of severe hypokalemia, a spot urine study demonstrated renal potassium wasting (Table 2). Given the presence of hypertension, metabolic alkalosis, and renal potassium wasting with severe hypokalemia, PA was strongly suspected [1, 4].

**Table 2:** Endocrine and renal potassium studies.

Parameter	Result	Diagnostic cutoff
Spot urine study		
Urine potassium (mmol/L)	20	—
Urine creatinine (mmol/L)	0.88	—
Urine potassium‐to‐creatinine ratio	22.73	>2.0 [4]
Screening test		
Plasma aldosterone (ng/dL)	33.58	—
Direct renin concentration (ng/L)	2.32	—
Aldosterone‐to‐renin ratio	14.47	>4.0 [1]
Confirmatory test		
Post‐captopril aldosterone (ng/dL)	47.82	>10 [1]
Post‐captopril direct renin (ng/L)	2.66	—

After appropriate washout of interfering medications, screening for PA was positive. Plasma aldosterone concentration was 33.58 ng/dL with a direct renin concentration of 2.32 ng/L, yielding an aldosterone‐to‐renin ratio (ARR) of 14.47 ng/dL per ng/L, exceeding the screening threshold of 4.0 ng/dL per ng/L. Subsequent confirmatory testing further supported the diagnosis: a captopril suppression test demonstrated a persistently elevated post‐captopril plasma aldosterone concentration of 47.82 ng/dL (diagnostic cutoff >10 ng/dL) with a concurrent direct renin concentration of 2.66 ng/L. These findings confirmed the diagnosis of PA (Table 2) [1].

**Figure 2:**
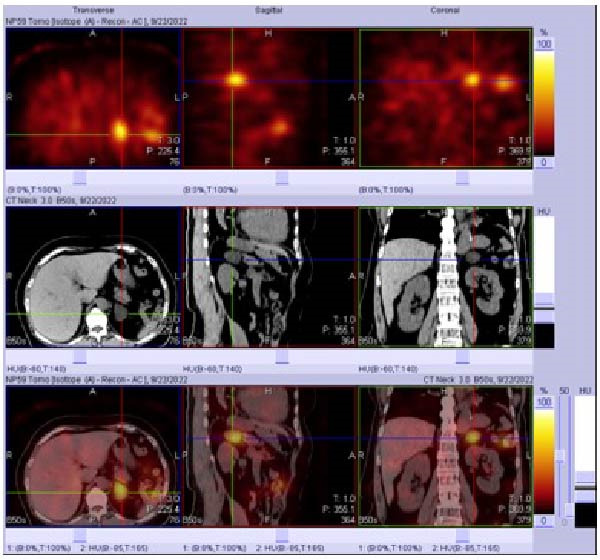
NP‐59 SPECT/CT demonstrating a left‐sided functioning adrenal adenoma. Fusion SPECT/CT imaging revealed focal accumulation of NP‐59 radiotracer in the left adrenal gland, consistent with a hyperfunctioning adrenal cortical adenoma.

**Figure 3:**
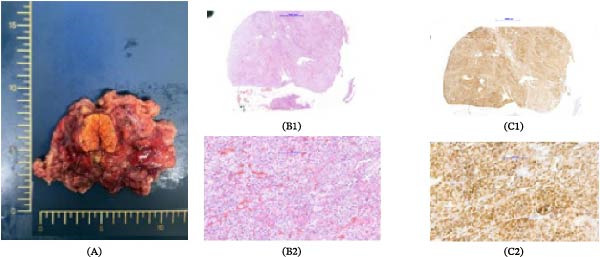
Gross and microscopic pathology of the aldosterone‐producing adenoma. (A) Gross examination of the resected left adrenal tumor, showing a yellow and well‐circumscribed mass with focal cystic degeneration. (B) Hematoxylin and eosin (H&E) staining. (B1) Low‐power view shows a well‐defined tumor composed of polygonal cells with eosinophilic cytoplasm. (B2) Higher magnification reveals tumor cells with prominent nucleoli and abundant cytoplasm. Scale bars: 5000 μm (B1) and 200 μm (B2). (C) Immunohistochemical staining for CYP11B2. (C1) Focal areas of positive staining observed within the lesion. (C2) Strong cytoplasmic CYP11B2 expression in tumor cells, supporting the diagnosis of aldosterone‐producing adenoma. Scale bars: 5000 μm (C1) and 200 μm (C2).

To subtype PA, adrenal CT revealed a 3.0 cm left adrenal mass with heterogeneous enhancement and cystic components. There was no evidence of excess production of other adrenal hormones. Baseline serum cortisol, 24‐h urinary vanillylmandelic acid (VMA), and catecholamines were within normal limits. Functional imaging with NP‐59 SPECT/CT demonstrated radiotracer uptake in the left adrenal gland (Figure 2), consistent with a functioning unilateral adenoma. The patient subsequently underwent laparoscopic left adrenalectomy, and the procedure was uneventful. Pathological examination revealed a 2.9 cm × 2.1 cm × 2.0 cm adrenal tumor. Hematoxylin and eosin (H&E) staining and immunohistochemistry for CYP11B2 confirmed the diagnosis of an aldosterone‐producing adenoma (Figure 3). Postoperatively, serum potassium stabilized within the normal range, around 4.3–5.0 mmol/L, and ARR decreased to 0.43 ng/dL per ng/L, indicating complete biochemical remission [5]. Her blood pressure also improved over the following months, dropping from 174/101 mm Hg preoperatively to 115/72 mm Hg at 15 months. The number of antihypertensive agents was reduced from three to two, indicating partial clinical remission [5].

## 3. Discussion

To the best of our knowledge, this is the first reported case in which DHS served as the initial manifestation of PA. The patient initially presented with axial neck extensor weakness, leading to suspicion of cervical neuromuscular pathology, before developing proximal limb muscle weakness, more characteristic of PA–related hypokalemic myopathy [3]. DHS is an uncommon clinical condition characterized by pronounced weakness of the cervical paraspinal muscles, resulting in correctable cervical flexion [6]. Although it is most commonly associated with neuromuscular disorders such as isolated neck extensor myopathy, Parkinson’s disease, myasthenia gravis, and amyotrophic lateral sclerosis [6], metabolic etiologies like hypokalemia are infrequently considered. A scant number of case reports have identified hypokalemia as a potentially reversible cause of DHS [7], highlighting the importance of including electrolyte disturbances in the differential diagnosis of patients presenting with acute neck extensor weakness. Early recognition of PA in such presentations is crucial to avoid misdiagnosis and to allow timely and definitive treatment.

Additionally, another notable clinical feature in our patient was hypokalemia‐induced rhabdomyolysis. Rhabdomyolysis is typically associated with hyperkalemia due to muscle breakdown. However, in rare instances, severe hypokalemia (typically below 2.5 mmol/L) can itself precipitate rhabdomyolysis [2]. Proposed mechanisms include impaired muscle perfusion due to capillary constriction, disruptions in glycogen metabolism, and abnormalities in transmembrane ion transport [2].

Hypokalemic rhabdomyolysis resulting from PA is infrequent, approximately 38 cases have been reported in the literature from 1976 to 2023 [2]. A previous systematic review identified several common features in such cases, including a predominance in younger individuals, a higher incidence among women, and a strong association with unilateral PA (reported in 93% of patients) [2]. Consistent with these findings, our patient, despite being older, was female and had a unilateral aldosterone‐producing adenoma. These observations further support the need for clinicians to consider PA as an underlying etiology in patients presenting with hypokalemic rhabdomyolysis, especially given the potential for surgical cure in unilateral forms [1].

## 4. Conclusion

To sum up, this case highlights two key teaching points. First, clinicians should broaden the differential diagnosis of DHS to include metabolic causes such as profound hypokalemia, which can even represent the initial manifestation of PA, as shown in our patient. Reliance solely on imaging without appropriate laboratory evaluation may lead to misdiagnosis. Second, in cases of hypokalemic rhabdomyolysis, it is crucial to investigate the underlying cause of hypokalemia rather than merely providing potassium supplementation. PA should be considered as a potential etiology, particularly in patients with otherwise unexplained or recurrent episodes.

Recognizing these atypical presentations facilitates timely diagnosis and definitive treatment, thereby preventing recurrent crises and reducing long‐term complications.

## Author Contributions

Ya‐Chen Kao was involved in data acquisition, literature review, and drafting of the manuscript. Yu‐Cheng Liang contributed to interpretation of the clinical data and assisted in drafting and revising the manuscript. Ye‐Fong Du was responsible for the clinical management of the patient. Horng‐Yih Ou contributed to study conception, supervision, and critical revision of the manuscript for important intellectual content.

## Funding

This research did not receive any specific grant from funding agencies in the public, commercial, or not‐for‐profit sectors.

## Disclosure

Horng‐Yih Ou had full access to all of the data in this study and takes complete responsibility for the integrity of the data and the accuracy of the data analysis. All authors participated in data interpretation, reviewed the manuscript, and approved the final version.

## Ethics Statement

This study was approved by the Institutional Review Board of National Cheng Kung University Hospital (Number A‐EC‐114‐021).

## Consent

Written informed consent was obtained from the patient for publication of this case report and any accompanying images.

## Conflicts of Interest

The authors declare no conflicts of interest.

## Supporting Information

Additional supporting information can be found online in the Supporting Information section.

## Supporting information


**Supporting Information** The CARE checklist for this case report is provided as supporting information.

## Data Availability

The data supporting the findings of this study are available from the corresponding author upon reasonable request, due to privacy and ethical restrictions.
